# Acceptability of Male Circumcision among College Students in Medical Universities in Western China: A Cross-Sectional Study

**DOI:** 10.1371/journal.pone.0135706

**Published:** 2015-09-21

**Authors:** Junjun Jiang, Jinming Su, Xiaobo Yang, Mingbo Huang, Wei Deng, Jiegang Huang, Bingyu Liang, Bo Qin, Halmurat Upur, Chaohui Zhong, Qianqiu Wang, Qian Wang, Yuhua Ruan, Li Ye, Hao Liang

**Affiliations:** 1 Guangxi Key Laboratory of AIDS Prevention and Treatment, School of Public Health, Guangxi Medical University, Nanning, Guangxi, 530021, China; 2 Guangxi Medical Research Center, Guangxi Medical University, Nanning, Guangxi, 530021, China; 3 Department of Microbiology, Biochemistry, and Immunology, Morehouse School of Medicine, Atlanta, Georgia, 30310, United States of America; 4 The First Affiliated Hospital, Chongqing Medical University, Chongqing, 400016, China; 5 School of Public Health, Xinjiang Medical University, Xinjiang, 830011, China; 6 School of Public Health, Chongqing Medical University, Chongqing, 400016, China; 7 National Center for STD Control, Chinese Center for Disease Control and Prevention, Nanjing, 210042, China; 8 State Key Laboratory for Infectious Disease Prevention and Control (SKLID), Collaborative Innovation Center for Diagnosis and Treatment of Infectious Diseases, Beijing, 102206, China; Fudan University, CHINA

## Abstract

**Background:**

Male circumcision (MC) has been shown to reduce the risk of female to male transmission of HIV. The goal of this survey was to explore MC’s acceptability and the factors associated with MC among college students in medical universities in western China.

**Methods:**

A cross-sectional study was carried out in three provinces in western China (Guangxi, Chongqing and Xinjiang) to assess the acceptability of MC as well as to discover factors associated with the acceptability among college students in medical universities. A total of 1,790 uncircumcised male students from three medical universities were enrolled in this study. In addition, 150 students who had undergone MC were also enrolled in the survey, and they participated in in-depth interviews.

**Results:**

Of all the uncircumcised participants (n = 1,790), 55.2% (n = 988) were willing to accept MC. Among those who accepted MC, 67.3% thought that MC could improve their sexual partners’ hygiene, 46.3% believed that HIV and sexually transmitted diseases (STDs) could be partially prevented by MC. The multivariable logistic regression indicates that MC’s acceptability was associated with three factors: the redundant foreskin (OR = 10.171, 95% CI = 7.629–13.559), knowing the hazard of having a redundant foreskin (OR = 1.597, 95% CI = 1.097–2.323), and enhancing sexual pleasure (OR = 1.628, 95% CI = 1.312–2.021). The in-depth interviews for subjects who had undergone MC showed that the major reason for having MC was the redundant foreskin (87.3%), followed by the benefits and the fewer complications of having MC done. In addition, most of these participants (65.3%) said that the MC could enhance sexual satisfaction.

**Conclusions:**

MC’s acceptance among college students in medical universities is higher than it is among other populations in western China. An implementation of an MC programme among this population is feasible in the future.

## Introduction

At present, the AIDS epidemic in China is still grim. By the end of September 2013, 434,000 people were reported to be living with HIV/AIDS, according to the Chinese Ministry of Health[1]. Currently, sexual transmission continues to be the primary mode of HIV-1 transmission in China, with 69.1% through heterosexual contact and 20.8% through homosexual contact in the newly reported cases[2]. Challenges in HIV/AIDS prevention and control remain critical in some regions, especially in six provinces: Yunnan, Guangxi, Henan, Sichuan, Xinjiang, and Guangdong, accounting for 75.8% of the national total [3]. Four of these high prevalence provinces are located in western China, where a poor economic environment, a widespread high-risk of sexual behaviour, and a lower level of public awareness of HIV/AIDS are believed to account for the high HIV/AIDS prevalence [4].

Randomized controlled trials (RCTs) in South Africa, Kenya, and Uganda have shown that male circumcision (MC) can reduce the risk of human immunodeficiency virus (HIV) transmission in heterosexual men by 50%–60% [5–7]. MC is also an important supplemental strategy for prevention of heterosexually contracted infections in men, and it is recommended by the WHO and UNAIDS [8]. Several other studies have also suggested that uncircumcised men have a higher risk of acquiring sexually transmitted diseases (STDs) including syphilis, gonorrhoea, and chlamydia, than do circumcised men [9, 10]. MC may be more effective in preventing or controlling HIV transmission in countries where HIV prevalence is high, the MC rate is low, and the predominant transmission is through heterosexual behaviours [11, 12].

For the HIV/AIDS epidemic, youth constitute a neglected group. While the global AIDS mortality decreased by 30%, the mortality of infected young people rose 50%, which is a significant increase in mortality during the past ten years [13]. The number of new infections in young students aged 14 to 25 years old has presented an annual increase, which has accounted for a rising proportion of new HIV/AIDS cases, ranging from 0.9% to 1.7% from 2008 to 2012 [14]. In addition, HIV/AIDS-reported cases in students have shown a rising trend from 0.96% in 2006 to 1.64% in 2011. Among them, the proportion of those aged 20 to 24 years old increased from 20.3% in 2006 to 49.0% in 2011[3]. All of these facts indicate that HIV/AIDS infections are trending toward the younger population, with the ratio of the disease contracted through sexual transmission on the rise among college students. College students are a high risk group for HIV infection as well as they are one of the focus populations for AIDS prevention [15]. They are prone to high-risk sexual behaviours because many college students are sexually active, have liberal attitudes towards sex, and are under social and personal pressure [16]. College students accounted for 55% of reported premarital sexual behaviour [17]. Furthermore, a study with 2,841 students participating found that 63.3% of college students have reported to begin having sex during their university education [18]. If college students are lacking in relevant sexual knowledge and prevention awareness, they will have a high risk of acquiring HIV.

Traditional prevention methods have not been as effective for HIV/AIDS prevention, thus new strategies, such as MC, are urgently needed to increase the effectiveness of HIV/AIDS prevention and control. MC has a high efficiency, fewer complications, and has a low cost measure for preventing HIV acquisition in males. In China, only about 5% of Chinese males have undergone MC, which is much lower than the overall rate of MC (30–34%) in the world [19, 20]. Investigations of MC acceptability have been conducted in several countries, including in sub-Saharan Africa, The United States, and Thailand [21–23], and MC is a very common in these areas. However, MC is not a common practice in China. It is a critical issue to promote MC in China.

In this study, we selected medical college students to investigate their HIV-1 knowledge, cognition, attitude, and willingness to accept, and the influencing factors for MC. We focused on medical college students for several reasons. First, medical students have professional medical knowledge and could become the pioneers of promoting an MC programme. Second, an investigation of medical students not only could improve this population’s MC knowledge, but it could also contribute to the increase of their MC acceptability. Third, we expect that medical college students could have a demonstration effect in receiving MC that might better develop the promotional material of circumcision among other populations, including non-medical students and other HIV-1 high-risk populations in China.

## Methods

### Study design and subjects

A cross-sectional survey was conducted in the form of face-to-face structured interviews in three universities in western China (Guangxi Medical University, Chongqing Medical University and Xinjiang Medical University) between June 2009 and November 2010. Participants were enrolled from their freshmen to their senior years of medical studies by a random cluster sampling method.

Meanwhile, a qualitative research was carried out through in-depth interviews. Male medical students in three universities who had undergone MC previously were recruited for this research. The recruitment was stopped when the addition of new subjects could not offer useful information. Eventually, though, a total of 150 students were recruited.

The subjects who were unable to provide voluntarily informed consent were excluded. The study was approved by the Ethics and Human Subjects Committee (EHSC) of the Guangxi Medical University.

### Questionnaires and data management

A 52-item questionnaire was designed with the main purpose of obtaining information on the acceptability of MC as an effective strategy to prevent HIV (i.e. ‘‘willingness to be circumcised”). The questionnaire has four sub-sections: demographic characteristics, general knowledge about MC and AIDS, willingness and reasons for accepting or refusing MC, and factors associated with the willingness to be circumcised. A few open-ended questions were asked. Most of the primary outcome variables were assessed by close-ended questions, such as “Do you know that MC can prevent AIDS and STDs?” with response categories of "Yes/No".

Twenty-three questions were asked to assess the participants’ knowledge about AIDS and MC, including 11 questions about general knowledge of AIDS, such as the pathway of HIV transmission and infection; and 12 questions were asked about MC, for example, the most suitable period and targeted population, and the advantages compared to the adverse effects after surgery [24].

For AIDS knowledge, we computed the average score among all of the interviewed subjects. The AIDS knowledge section of the questionnaire was scored with one point given to each correct answer, while wrong answers or answers that that showed the respondent did not know were scored as zero. Respondents’ willingness to accept MC was assessed with the question "Do you want to be circumcised to prevent HIV?", and the response categories were "definitely willing", "probably willing", "definitely not willing" and "probably not willing". For analysis, we dichotomized the groups of "definitely willing" and "probably willing" into a single variable of "willingness to be circumcised (WTC)", and the groups of "definitely not willing" and "probably not willing" were categorized as "unwillingness to be circumcised (non-WTC)". To assess reasons for accepting or refusing MC, 12 open-/close-ended questions were asked for the advantages and disadvantages of MC and surgery costs, etc. Data were collected by trained Research Assistants (RAs). After the subjects provided their written informed consents to participate in the study, RAs conducted the detailed interviews following the structured guidelines.

We carried out the qualitative research by face-to-face interviews. The research contents include the reasons for MC, complications of MC, postoperative feelings, and the relationship between the foreskin and HIV.

### Analysis

All the data were entered into EpiData software (EpiData 3.1 for Windows; The EpiData Association Odense, Denmark) and analysed using SPSS for Windows Version 16.0 (SPSS, Chicago, IL, USA). Descriptive statistics were generated for each of the variables, corresponding to specific questions in the survey, including general characteristics and reasons for accepting or refusing MC. To compare basic characteristics between the two groups, we used a chi-squared test. We performed a multivariate logistic regression analysis to identify factors associated with MC’s acceptability. Variables that showed statistically significant associations (p<0.05) with the willingness to be circumcised were included in a univariate analysis. All statistical tests were two sided with a significant level of p<0.05.

## Results

### Demographic characteristics

A total of 2,022 subjects were interviewed, and 1,790 completed questionnaires (response rate: 88.5%). As shown in Table 1, of the respondents (n = 1790), 26.3% (n = 470) were from Guangxi Medical University, 30.4% (n = 545) from Chongqing Medical University, and 43.3% (n = 775) from Xinjiang Medical University. Of the respondents, 34.0% were freshmen, 48.3% were majoring in clinical medicine, 89.3% were of Han ethnicity, 89.8% did not smoke, and 90.3% did not drink (Table 1).

**Table 1:** Demographic characteristics of subjects (n = 1790).

Variables	No.	Percent (%)
Total samples	1790	100.0
**Provinces**		
Guangxi	470	26.3
Chongqing	545	30.4
Xinjiang	775	43.3
**Grades**		
Freshman	609	34.0
Sophomore	484	27.0
Junior	404	22.6
Senior	293	16.4
**Major**		
Clinical Medicine	865	48.3
Preventive Medicine	190	10.6
Other majors	735	41.2
**Ethnic group**		
Han	1598	89.3
Other Minorities	192	10.7
**Smoking**		
Yes	182	10.2
No	1608	89.8
**Drinking**		
Yes	173	9.7
No	1617	90.3

### Knowledge of AIDS and MC among the medical students

For all respondents, the average score of AIDS knowledge was 6.04 (out of 10) (Table 2). Of them, 77.8% (n = 1,393) had less than average scores; however, the difference between the non-WTC group (79.4%) and the WTC group (75.9%) had no statistical significance (P>0.05).

**Table 2:** Knowledge of AIDS and MC among the medical students.

Variables	n	Percent (%)
**Score of AIDS knowledge**		
Less than average score	1393	77.8
Average score or higher	397	22.2
**Do you know what MC is?**		
Yes	1459	81.5
No	331	18.5
**Do you know that MC can prevent penile inflammation and cancer?**		
Yes	923	51.6
No	867	48.4
**Do you know that MC can prevent AIDS and STDs?**		
Yes	537	30
No	1253	70
**Do you know that MC can improve sexual partners’ hygiene?**		
Yes	960	53.6
No	830	46.4
**Do you know that MC can enhance sexual pleasure in the future?**		
Yes	726	40.6
No	1064	59.4
**Do you know MC can improve penile appearance?**		
Yes	238	13.3
No	1552	86.7
**Do you know the hazard of redundant foreskin?**		
Yes	1637	91.5
No	153	8.5
**Do you feel that your foreskin is redundant or too long?**		
Yes	538	30.1
No	1252	69.9
**Did you have sexual intercourse in the past year**		
Yes	245	13.7
No	1545	86.3

The average score of AIDS knowledge is 6.04

Of all respondents (n = 1,790), 81.5% (n = 1459) knew what MC is, and 51.6% (n = 923) understood that MC could prevent penile inflammation and cancer, but only 30% (n = 537) knew that MC could prevent AIDS and STDs. Furthermore, 53.6% (n = 960) knew that MC could improve their sexual partners’ hygiene, 59.4% (n = 1064) knew that MC could enhance sexual pleasure in the future, 86.7% (1,552) did not know that MC could improve penile appearance, 91.5% (n = 1637) knew the hazards of having a redundant foreskin, but 69.9% (n = 1252) did not feel that the foreskin was redundant or too long, and 86.3% (n = 1545) had not had sexual intercourse in the past year.

### Acceptability of MC and reasons to accept or refuse MC

Of all the respondents (n = 1,790), 55.2% (n = 988) were willing to accept MC in order to prevent HIV/STD. In Guangxi Medical University, 53.8% (253/470) of the respondents were willing to accept MC. In Chongqing and Xinjiang Medical University, the acceptance rates were 55.0% (300/545) and 56.1% (435/775), respectively. The difference among the three universities has no statistical significance (*p* = 0.729). As shown in Table 3, of those who were willing to accept MC (n = 988), 67.3% thought that MC could improve their sexual partners’ hygiene, 67.2% had a self-reported redundant foreskin, 67.2% thought that MC could help prevent penile cancer, 46.3% believed that HIV and STDs could be partially prevented by MC, 53.3% felt that MC could enhance sexual pleasure, and 20% though that MC could improve penile appearance. Of those who refused MC (n = 802), 4.2% (n = 595) believed it would not be necessary or effective for them, 34.7% were concerned about the potential danger associated with surgery, 18.8% were worried about the reduction of sexual ability, and 14.8% worried about the expensive cost of MC surgery.

**Table 3:** Reasons to accept or refuse MC among the medical students.

Reasons	n	Percent (%)
**Willing to accept MC**	988	100.0
Improve partners’ hygiene	665	67.3
Redundant foreskin	664	67.2
Prevention of penile cancer	664	67.2
Enhance sexual pleasure	527	53.3
Protection against HIV and STDs	457	46.3
Better penile appearance	198	20
Traditional or religious reason	34	3.4
**Refuse MC**	802	100
Not necessary or not effective	595	74.2
Concern about potential danger associated with surgery	278	34.7
Concern about reducing sexual ability	151	18.8
Concern about expensive surgery cost	119	14.8

In order to explore the reasons for these participants’ unwillingness to accept MC, we explained MC’s benefits to participants who were unwilling to be circumcised. After this, 17% (136/802) of these participants changed their mind when they were told that MC had few surgery-related complications. If MC surgery could be provided for free, 15.6% (125/802) indicated they would accept MC.

### Factors associated with acceptability of MC

The univariate analysis identified nine potential factors that were significantly associated with MC preferences (p<0.05) (Table 4), including drinking, knowing what MC is, knowing that MC can prevent penile inflammation and cancer, knowing that MC can prevent AIDS and STDs, knowing that MC can improve their sexual partners’ hygiene, knowing that MC can enhance sexual pleasure in the future, knowing that MC can improve penile appearance, knowing the hazard of having a redundant foreskin, and feeling that the foreskin is redundant or too long.

**Table 4:** Factors associated with the willingness to be circumcised.

Variables	WTC group n = 988 (%)	Non-WTC group n = 802 (%)	χ^2^	*P* value
**Universities**			0.633	0.729
Guangxi Medical University	253(25.6)	217(27.1)		
Chongqing Medical University	300(30.4)	245(30.5)		
Xinjiang Medical University	435(44.0)	340(42.4)		
**Grades**			7.357	0.061
Freshman	316(32.0)	293(36.5)		
Sophomore	261(26.4)	223(27.8)		
Junior	242(24.5)	162(20.2)		
Senior	169(17.1)	124(15.5)		
**Major**			4.392	0.111
Clinical Medicine	495(50.1)	370(46.1)		
Preventive Medicine	109(11.0)	81(10.1)		
Other major	384(38.9)	351(43.8)		
**Ethnic group**			0.584	0.445
Han	887(89.8)	711(88.7)		
Other Minorities	101(10.2)	91(11.3)		
**Smoking**			0.059	0.808
Yes	102(10.3)	80(10.0)		
No	886(89.7)	722(90.0)		
**Drinking**			4.724	0.030
Yes	109(11.0)	64(8.0)		
No	879(89.0)	738(92.0)		
**Score of AIDS knowledge**			2.995	0.084
Less than average score	784(79.4)	609(75.9)		
Average score or higher	204(20.6)	193(24.1)		
**Knowing what MC is**			26.059	0.000
Yes	847(85.7)	612(76.3)		
No	141(14.3)	190(23.7)		
**Knowing that MC can prevent penile inflammation and cancer**			9.580	0.002
Yes	542(54.9)	381(47.5)		
No	446(45.1)	421(52.5)		
**Knowing that MC can prevent AIDS and STDs**			13.633	0.000
Yes	332(33.6)	205(25.6)		
No	656(66.4)	597(74.4)		
**Knowing that MC can improve sexual partners’ hygiene**			28.613	0.000
Yes	586(59.3)	374(46.6)		
No	402(40.7)	428(53.4)		
**Knowing that MC can enhance sexual pleasure in the future**			48.954	0.000
Yes	473(47.9)	253(31.5)		
No	515(52.1)	549(68.5)		
**Knowing that MC can improve penile appearance**			16.067	0.000
Yes	160(16.2)	78(9.7)		
No	828(83.8)	724(90.3)		
**Knowing the hazard of redundant foreskin**			17.275	0.000
Yes	928(93.9)	709(88.4)		
No	60(6.1)	93(11.6)		
**Feeling that foreskin is redundant or too long**			340.656	0.000
Yes	475(48.1)	63(7.9)		
No	513(51.9)	739(92.1)		
**having sexual intercourse in the past year**			1.155	0.283
Yes	143(14.5)	102(12.7)		
No	845(85.5)	700(87.3)		

Note: WTC: willingness to be circumcised. Non-WTC: no willingness to be circumcised.

Compared to the non-WTC group, the WTC group possessed better knowledge about MC (Table 4). In WTC group, 85.7% knew what MC is, compared to 76.3% in the non-WTC group (P < 0.05); 54.9% knew that MC can prevent penile inflammation and cancer, compared to 47.5% in the non-WTC group (p<0.05). Overall 33.6% (n = 332) knew that MC could prevent AIDS and STDs, with more subjects in the WTC group than in the non-WTC group (33.6% versus 25.6%, p<0.05). In the WTC group, more subjects knew that MC could improve their sexual partners’ hygiene than did those in the non-WTC group (59.3% versus 46.6%, p<0.05), and more knew about the hazards of having a redundant foreskin (93.9% versus 88.4%, p<0.05) (Table 4).

The multivariable logistic regression model, including the abovementioned nine significant variables, identified three factors that were associated with MC’s acceptability: having a redundant foreskin (yes versus no, OR = 10.171, 95% CI = 7.629–13.559), the hazards of having a redundant foreskin (yes versus no, OR = 1.597, 95% CI = 1.097–2.323), and knowing that MC can enhance sexual pleasure (yes versus no, OR = 1.628, 95% CI = 1.312–2.021) (Table 5).

**Table 5:** Multivariate analysis of willingness to be circumcised among 1790 subjects who had face-to-face interview.

Variables	*Adjusted OR (95% CI)*	*P* value
**Do you feel that your foreskin is redundant or too long**		
No	1.00	
Yes	10.171(7.629–13.559)	0.000
**Do you know the hazard of redundant foreskin?**		
No	1.00	
Yes	1.597(1.097–2.323)	0.014
**Do you know that MC can enhance sexual pleasure in the future?**		
No	1.00	
Yes	1.628(1.312–2.021)	0.000

### The in-depth interviews

In the qualitative research, 150 subjects were recruited, including 104 undergraduates and 46 graduate students. Fifty subjects came from Guangxi, 45 from Chongqing and 55 from Xinjiang. The mean age was 23, which varied between 19 and 30 years old. Most of them were majoring in clinical medicine.

The major reason for having MC was having the redundant foreskin (87.3%). Most of the interviewees (74.7%) knew the complications (edema, pain, etc.) and MC’s benefits (maintaining penile hygiene, enhancing sexual pleasure, etc.). Their postoperative complications were few, and most of them only had tiny pain and were a little bit swollen. In addition, the interviewees didn’t like others to know that they had undergone MC. However, even when the others knew the fact, the interviewees did not feel any discrimination. Furthermore, half of the interviewees (65.3%) who were sexually active believed the circumcision surgery could enhance their sexual satisfaction. However, most of the medical students who had undergone MC did not know that MC could effectively prevent HIV. When we explained the epidemiological evidence and molecular mechanism of MC’s prevention of HIV, they accepted this view and were willing to volunteer for promoting MC in regions with high incidences of AIDS.

## Discussion

The study is the first one to investigate MC’s acceptability and related factors among medical college students in China. Our investigation showed a higher overall MC acceptance rate (55.2%) among this population, compared to other populations in China, such as 44.6% among the general population [25], 45.2% among the drug abusing population[26], 37.3% among the male rural-to-urban migrants [27], and 40.6% among young people of Yi nationality in Sichuan province[28]. However, the rate was still lower than the reported rates in other countries, such as 65% in sub-Saharan Africa [21] and 87.7% among the population of men who have sex with men (MSM) in the USA [22]. Apparently, the higher acceptance rate among medical students than among the general and other populations in China is due to their medical background, which also suggests that the implementation of an MC programme among this population would be more feasible than it would be among the general and other populations in China in the future.

In the multivariable analysis, the factors associated with the acceptability of MC included the redundant foreskin, the hazard of having a redundant foreskin and the knowledge that MC can enhance sexual pleasure. Several previous studies also reported partially similar factors associated with the acceptance of MC, including the presence of phimosis, maintaining penile hygiene, MC knowledge, increasing sexual pleasure, and preventing HIV and other sexually transmitted infections [29–31]. In this study, we also found that the reasons among medical students in China for being willing to have an MC included improvement of partners’ hygiene (67.3%), having a redundant foreskin (67.2%) and the prevention of penile cancer (67.2%), while more than half of the subjects (74.2%) in the non-WTC group believed that MC was not necessary or not effective. These results are roughly consistent with those of previous studies [21, 32]: in Malawi, a country with a high HIV prevalence and a low prevalence of MC, for example, the acceptance of MC included hygiene, reduced risk of STDs, religion, medical conditions, and enhanced sexual pleasure.

Obviously, religious tradition is a strong factor affecting the acceptability of MC. Several previous studies found that other populations had higher acceptability among residents, IDUs and others in Xinjiang province of China [25, 26], where many Muslims were centralized and circumcised as a religious tradition. Iliyasu’s survey also showed that almost all respondents, which consisted of university students in northern Nigeria (98.1%), reported being circumcised, most circumcisions were performed for religious tradition (79.2%), although only 38% of the respondents were aware of the role of MC in reducing HIV acquisition in heterosexual males [33]. However, our investigation indicates that medical students in the three universities in Xinjiang (56.1%), Chongqing (55.0%) and Guangxi (53.8%) have a similar level of accepting MC. The results showed no significant difference among these three universities. The main reason for this is that the college students in these three universities are mainly from the whole country. Nearly 90% of them are Ethnic Han, and only 10% belong to minorities, with only a few Muslim students.

Our investigation indicates that participants who had better knowledge about MC, including the fact that MC can prevent penile inflammation and cancer, prevent AIDS and STDs, improve sexual partners’ hygiene, enhance sexual pleasure and improve penile appearance, were more likely to accept MC. These results are consistent with earlier findings in the Dominican Republic [34], Africa, the United States and among the general public in China [25]. Supportively, some of subjects who refused to accept MC at first changed their minds after receiving information in the questionnaire about the benefits of circumcision. Thus, we believe increased knowledge about the protective effects of MC would have an impact on participants’ attitudes towards MC’s acceptability. Medical students are more likely to accept MC if they are aware of the association between MC and AIDS prevention, which is new information that has not yet been integrated into public health education campaigns. This information would encourage the public to consider MC as one of the protective measures against HIV prevention in addition to other protective measures.

The in-depth interviews showed that circumcised medical students had medical knowledge, health preventive awareness, circumcision-related knowledge, and so on. However, most interviewees did not know why MC could prevent HIV infection. When investigators explained the biological mechanism of MC’s preventing HIV, the interviewees accepted the view, and most of them expressed their willingness to be volunteers to promote an MC programme. So the circumcised medical students could play an important role in promoting MC, with the combination of better MC knowledge and their own experience.

Several limitations should be taken into account in this study. First, the lack of investigation of non-medical student weakens the scientific significance of this study. Limited by manpower and financial resources, this study only enrolled medical college students to investigate. Nevertheless, with the comparison to the situations in other populations, such as the general population, drug abusers, the male rural-to-urban migrants, young people, etc., our investigation of medical students has quite an important value for the promotion of an MC programme in China, due to the professional medical knowledge of the population as well as their potential to become pioneers of promoting an MC programme. Second, our study used convenience sampling, which may lead to selection bias. Those who participated in the study were perhaps more concerned about their health and more interested in the topic. Third, we only selected three medical universities, all of which were in western provinces in China, and therefore the sampling is not sufficiently representative. To have a more accurate estimate of the whole region, expanding the study to involve more medical universities and a larger sample size would be necessary. Finally, the collected information was mainly based on reported behaviours and characteristics, such as the self-reported long foreskin, without any clinical examination or other confirmation.

In summary, the study indicates a higher MC acceptance rate among medical students in western China, compared to those among the general or other populations in China. Three factors that were identified as predictors of willingness to accept MC can be used to design a programme to promote MC among this population in China. However, the educational information about the protective effects of MC against HIV/AIDS spread needs to be strengthened in the future, because this investigation did not find this information to be an associated factor for willingness to accept MC.

## Supporting Information

S1 File The raw data of MC among medical university. (XLS)

S2 File The data's description of MC among medical university. (DOC)
