# Supplementary material for: Acceptability of Male Circumcision among College Students in Medical Universities in Western China: A Cross-Sectional Study
Source: PLoS One. 2015 Sep 21;10(9):e0135706. doi: 10.1371/journal.pone.0135706 (PMC4577094; doi:10.1371/journal.pone.0135706)
Supplement: S2 File — (DOC) [file pone.0135706.s002.doc]

**Description of Data**

- **Number:** A total of 1,790 subjects completed the questionnaires
- **Group:** 1=willingness to be circumcised (WTC group); 2=unwillingness to be circumcised (Non-WTC group)
- **Provinces/university:** 1=Guangxi/ Guangxi Medical University; 2=Chongqing/ Chongqing Medical University; 3= Xinjiang/ Xinjiang Medical University
- **Grades:** 1= Freshman; 2= Sophomore; 3=Junior; 4= Senior
- **Major**: 1= Clinical Medicine; 2= Preventive Medicine; 3=Other major
- **Ethnic group:** 1=Han; 2= Other Minorities
- **Smoking:** 1=Yes; 0=No
- **Drinking:** 1=Yes; 0=No
- **Question of AIDS knowledge 1-10:** 1= correct answer; 0= wrong answers or did not know the answers
- **Score of AIDS knowledge:** the total score of AIDS knowledge including the question 1-10
- **Total score VS average score:** 1= Total score < average score (6.4); 2= Total score > average score (6.4)
- **Do you know what MC is:** 1=Yes; 0=No
- **Do you know that MC can prevent penile inflammation and cancer:** 1=Yes; 0=No
- **Do you know that MC can prevent AIDS and STDs:** 1=Yes; 0=No
- **Do you know that MC can improve sexual partners’ hygiene:** 1=Yes; 0=No
- **Do you know that MC can enhance sexual pleasure in the future:** 1=Yes; 0=No
- **Do you know MC can improve penile appearance:** 1=Yes; 0=No
- **Do you know the hazard of redundant foreskin:** 1=Yes; 0=No
- **Do you feel that your foreskin is redundant or too long:** 1=Yes; 0=No
- **Did you have sexual intercourse in the past year:** 1=Yes; 0=No
- **Reasons to accept MC among the medical students**

| **Improve partners’ hygiene:** 1=Yes; 0=No |
| --- |
| **Redundant foreskin:** 1=Yes; 0=No |
| **Prevention of penile cancer:** 1=Yes; 0=No |
| **Enhance sexual pleasure:** 1=Yes; 0=No |
| **Protection against HIV and STDs:** 1=Yes; 0=No |
| **Better penile appearance:** 1=Yes; 0=No |
| **Traditional or religious reason:** 1=Yes; 0=No |

- **Reasons to refuse MC among the medical students**

| **Not necessary or not effective: :** 1=Yes; 0=No |
| --- |
| **Concern about potential danger associated with surgery:** 1=Yes; 0=No |
| **Concern about reducing sexual ability:** 1=Yes; 0=No |
| **Concern about expensive surgery cost:** 1=Yes; 0=No |
